# Vesicular stomatitis virus expressing interferon-β is oncolytic and promotes antitumor immune responses in a syngeneic murine model of non-small cell lung cancer

**DOI:** 10.18632/oncotarget.5320

**Published:** 2015-09-28

**Authors:** Manish R. Patel, Blake A. Jacobson, Yan Ji, Jeremy Drees, Shaogeng Tang, Kerry Xiong, Hengbing Wang, Jennifer E. Prigge, Alexander S. Dash, Andrea K. Kratzke, Emily Mesev, Ryan Etchison, Mark J. Federspiel, Stephen J. Russell, Robert A. Kratzke

**Affiliations:** ^1^ Department of Medicine, Division of Hematology, Oncology, and Transplantation, University of Minnesota Medical School, Minneapolis, MN, USA; ^2^ Department of Surgery, University of Minnesota Medical School, Minneapolis, MN, USA; ^3^ Mayo Clinic, Department of Molecular Medicine, Rochester, MN, USA

**Keywords:** oncolytic virus, NSCLC, interferon-β, VSV, Treg

## Abstract

Vesicular stomatitis virus (VSV) is a potent oncolytic virus for many tumors. VSV that produces interferon-β (VSV-IFNβ) is now in early clinical testing for solid tumors. Here, the preclinical activity of VSV and VSV-IFNβ against non-small cell lung cancer (NSCLC) is reported. NSCLC cell lines were treated *in vitro* with VSV expressing green fluorescence protein (VSV-GFP) and VSV-IFNβ. VSV-GFP and VSV-IFNβ were active against NSCLC cells. JAK/STAT inhibition with ruxolitinib re-sensitized resistant H838 cells to VSV-IFNβ mediated oncolysis. Intratumoral injections of VSV-GFP and VSV-IFNβ reduced tumor growth and weight in H2009 nude mouse xenografts (*p* < 0.01). A similar trend was observed in A549 xenografts. Syngeneic LM2 lung tumors grown in flanks of A/J mice were injected with VSV-IFNβ intratumorally. Treatment of LM2 tumors with VSV-IFNβ resulted in tumor regression, prolonged survival (*p* < 0.0001), and cure of 30% of mice. Intratumoral injection of VSV-IFNβ resulted in decreased tumor-infiltrating regulatory T cells (T_reg_) and increased CD8^+^ T cells. Tumor cell expression of PDL-1 was increased after VSV-IFNβ treatment. VSV-IFNβ has potent antitumor effects and promotes systemic antitumor immunity. These data support further clinical investigation of VSV-IFNβ for NSCLC.

## INTRODUCTION

Oncolytic viruses are emerging as an effective treatment strategy for many tumor types [1, 2]. These viruses selectively infect and subsequently lyse cancer cells, while remaining relatively inert to most normal cells. Vesicular stomatitis virus (VSV) is a single-stranded RNA virus of the *Rhabdoviridae* strain. Although not a known human pathogen, VSV can cause limited illness in livestock and encephalitis in mice. As it is not a human pathogen, outside of livestock workers and laboratory workers, most people have never been exposed to VSV and harbor no pre-existing immunity to the virus making it an attractive choice for therapeutic application. Preclinical studies have shown that VSV is a potent oncolytic virus for many tumor types including lung cancer [3]. The tumor-specific tropism of VSV is largely based upon a defect in the type I interferon (IFN) response of many tumor tissues [4]. IFN activation in normal tissues rapidly thwarts viral replication, but in cancers, viral replication proceeds unabated and results in cell lysis. The clinical application of VSV has been limited by the potential for neurotoxicity previously observed in mice. In light of this, the engineering of recombinant VSV to express interferon-β (VSV-IFNβ) was undertaken to overcome this limitation. This strategy is based upon the notion that production of IFNβ during viral replication would strengthen the antiviral type I IFN response in most non-cancerous tissues [5–7]. Preclinical testing of this strategy showed that VSV-IFNβ retained oncolytic activity and was safely delivered to mice without observed neurotoxicity in mesothelioma and hepatoma models [5, 7]. Currently, a phase I clinical trial is evaluating the safety of intratumoral VSV-IFNβ for patients with hepatoma (NCT01628640). Parental VSV previously was shown to replicate in non-small cell lung cancer (NSCLC) cells [3]. Experiments herein demonstrate that VSV-IFNβ also has oncolytic activity against NSCLC *in vitro* and *in vivo*. Moreover, in an immune competent syngeneic murine model of NSCLC, VSV-IFNβ induces antitumor immune responses and has potent antitumor activity.

## RESULTS

### VSV-hIFNβ has oncolytic activity against NSCLC cell lines

**Figure 1:**
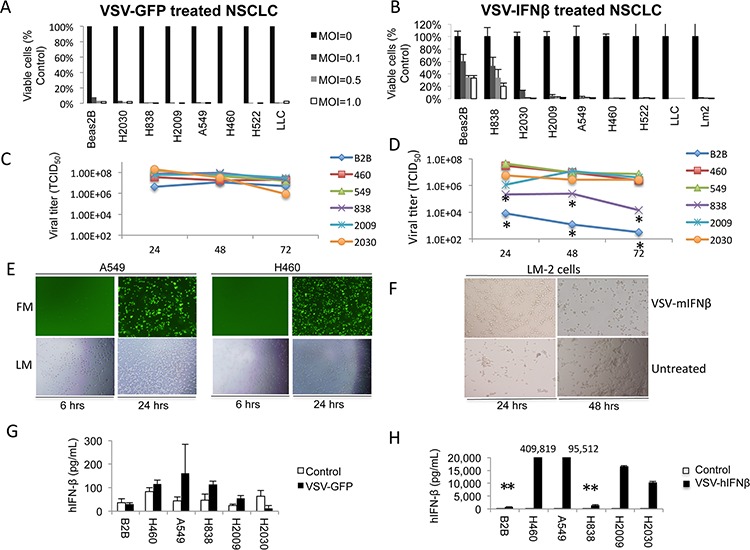
VSV-GFP and VSV-hIFNβ are cytotoxic to NSCLC cells **A.** Human NSCLC cell lines and Beas2B cells (control) were infected with VSV-GFP at the indicated MOI. Cell viability was determined after 72 hours by trypan blue exclusion. **B.** Human and murine NSCLC cell lines and Beas2B cells were infected with VSV-hIFNβ and VSV-mIFNβ, respectively, at the indicated MOI. LLC and LM2 are murine NSCLC lines; all other lines are human NSCLC. Cell viability was determined after 72 hours by trypan blue exclusion. **C** and **D.** Viral titer was determined by collecting supernatant from NSCLC cell lines and Beas2B cells treated *in vitro* with an MOI of 0.1. Supernatant was collected daily after infection with either VSV-GFP (C) or VSV-hIFNβ (D) * indicates statistically significant result comparing Beas2B and H838 to the rest of the NSCLC cell lines. **E.** Representative fluorescence and light micrographs of NSCLC cells infected with VSV-GFP at 6 and 24 hours. **F.** Light micrographs showing cytopathic effect of VSV-mIFNβ against murine LM2 cells after infection. **G** and **H.** Production of human IFNβ was determined by collecting supernatant from NSCLC cell lines and Beas2B cells treated *in vitro* with an MOI of 0.1. Supernatant was collected at 48 hours after infection with either VSV-GFP (G) or VSV-hIFNβ (H) and tested for the presence of hIFNβ by ELISA, and ** signifies *p* < 0.0001 comparing Beas2B and H838 to the other NSCLC cell lines. Numbers above the bar graphs indicate the hIFNβ level in the supernatant (in pg/mL). Error bars indicate standard deviation.

The cytotoxic effects of VSV-hIFNβ and VSV expressing green fluorescence protein (VSV-GFP) were compared among a panel of human and murine NSCLC cell lines and the non-malignant immortalized human lung bronchial epithelial cell line, Beas-2B. Near complete oncolysis was observed 72 hours after NSCLC cells and Beas-2B cells were exposed to VSV-GFP at all tested viral concentrations (Figure 1A). Even at a low multiplicity of infection (MOI) of 0.02, oncolysis among H2009, A549, and H460 cells was observed after exposure to VSV-GFP (data not shown). In contrast, VSV-hIFNβ was more cytotoxic to murine and human NSCLC cell lines than Beas-2B cells (Figure 1B). However, H838 cells were resistant to oncolysis even at higher MOI. Viral titers from supernatants of infected cells showed that viral replication correlated with cytoxic effects (Figure 1C and 1D). Viral titers were similar among NSCLC cells treated with VSV-GFP, however, treatment of Beas2B and H838 with VSV-hIFNβ resulted in several logs lower viral titer compared to the sensitive NSCLC cell lines. Cytopathic effect occurs maximally at 24 hours on visual inspection of infected cells as shown in Figure 1E and 1F. Of note, most of these cell lines are *K-ras* mutated except H838 and H522. H522 was highly permissive of VSV-hIFNβ suggesting that *K-ras* mutation is not necessary for viral replication. However, it cannot be assumed based on these data that VSV-hIFNβ will be oncolytic for all NSCLC subtypes, particularly those with other activating driver mutations.

The normal innate response to viral infection involves production of type I IFN that sets off a cascade of events designed to prevent viral spread [8]. Therefore, to determine the effect of VSV infection on IFNβ production, we collected supernatant of NSCLC cells and Beas-2B cells treated *in vitro* with an MOI of 0.1. Levels of secreted human IFNβ were measured from supernatants 48 hours after treatment with VSV-GFP or VSV-hIFNβ. VSV-GFP treatment resulted in slight increases in secreted IFNβ in H838 and H2009 cells (*p* < 0.05), but no statistically significant increase in the other cell lines was observed compared to untreated control cells (Figure 1G) demonstrating that most NSCLC cells have defects in IFNβ production upon viral infection as has been previously shown [9]. In contrast, VSV-hIFNβ treatment resulted in markedly increased secretion of hIFNβ for each cell line as compared to VSV-GFP treated cells (Figure 1H). The resistant H838 cell line and non-malignant Beas-2B cell line produced very little hIFNβ compared to the other NSCLC cell lines (*p* < 0.0001 for each cell line compared to Beas2B and H838). From these results, we conclude that most IFNβ production in VSV-hIFNβ-treated cells reflects viral transgene expression as a result of viral replication.

### NSCLC cells have defects in the IFN response to VSV-IFNβ

**Figure 2:**
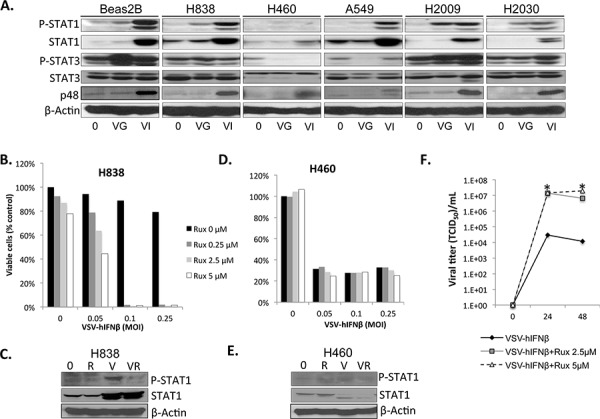
NSCLC cells are defective in IFN response to viral infection NSCLC cells were infected with VSV-GFP and VSV-hIFNβ, and cell lysates were prepared after 24 hours of infection at an MOI of 0.1. **A.** Signaling proteins in the IFN response were assayed by immunoblot. β-actin was used as a loading control. 0, untreated; VG, VSV-GFP; VI, VSV-hIFNβ. **B–E.** H838 cells and H460 cells grown in 96-well plates were treated with VSV-hIFNβ alone at indicated MOI or in combination with ruxolitinib at indicated doses. Viable cells were assayed 72 hours after treatment (B and D). Western blots for p-STAT1 were done in H838 cells and H460 cells (C and E) to demonstrate inhibition of p-STAT1 in H838 cells after ruxolitinib treatment. 0 = untreated, R = ruxolitinib 250 nM V = VSV-hIFNβ MOI 0.1, VR = combination VSV-hIFNβ and ruxolitinib. **F.** Supernatants from cells treated in parallel to B) were collected and assayed for viral titer at 24 and 48 hours post-infection. Data are expressed as TCID50/mL. * denotes *p* < 0.01 compared to VSV-hIFNβ alone.

Production of IFNβ by VSV-IFNβ appeared to attenuate viral oncolysis in control Beas-2B cells and NSCLC H838 cells as both of these cell lines were sensitive to VSV-GFP, but not the other NSCLC cell lines. Therefore, it was hypothesized that defects in the IFN signaling pathway might play a role in the sensitivity of NSCLC cells to VSV-hIFNβ. Therefore, we assessed the IFN signaling pathway in the panel of NSCLC cell lines (Figure 2A). H460, the most sensitive cell line to VSV-hIFNβ, exhibited a severely blunted induction of phosphorylation of STAT1 and STAT3, and little induction of p48 upon exposure to either VSV-GFP or VSV-hIFNβ compared to H838 cells and non-malignant Beas2B cells. All cell lines showed induction of endogenous STAT1 expression after VSV-hIFNβ but not after VSV-GFP treatment consistent with the effect of type I IFNβ on STAT1 expression seen previously in NSCLC cells [10]. JAK1/2 inhibition by ruxolitinib exposure directly upstream of STAT1 inhibits VSV-hIFNβ mediated STAT1 phosphorylation and re-sensitizes H838 cells to the VSV-hIFNβ (Figure 2B & 2C). Ruxolitinib alone induced very little cytotoxicity to H838 cells, but in combination with VSV-IFNβ resulted in near complete oncolysis. This correlated with nearly 3 logs higher viral titer measured in supernatants from combination treatment compared to VSV-IFNβ alone (Figure 2F). In contrast, in H460 cells with minimal inducement of STAT1, the addition of ruxolitinib did not have any effect on oncolysis or on STAT1 phosphorylation, as these cells were already robustly sensitive to VSV-hIFNβ (Figure 2D & 2E). A549 cells induce STAT1 phosphorylation, however, the p48 induction is blunted. H2009, and H2030 cells that are sensitive to VSV-hIFNβ also induce STAT1 phosphorylation and p48 expression after treatment (Figure 2A) in spite of their sensitivity to VSV-hIFNβ. Therefore, though intact JAK/STAT signaling mediates resistance in H838, intact STAT1 signaling is not sufficient to mediate resistance in other cell lines.

**Figure 3:**
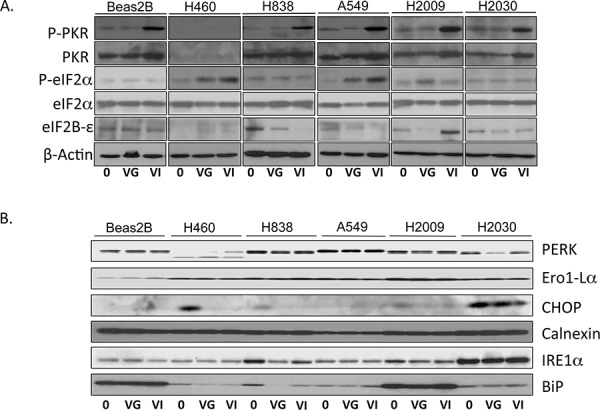
Western blot of PKR/eIF2α and ER stress pathway NSCLC cells were infected with VSV-GFP and VSV-hIFNβ at an MOI of 0.1. Lysates were prepared 24 hours following infection and assayed by immunoblot. **A.** Immunoblot of PKR and eIF2 pathway. **B.** Immunoblot of ER stress proteins. 0, untreated; VG, VSV-GFP; VI, VSV-hIFNβ. β-Actin was used as a loading control. The β-Actin in A) was from the same lysates as the immunoblots in B).

Dysfunctional PKR activity and its downstream effects mediated through eIF2α impacting translational control have been posited as a major mechanism of tumor tropism for a variety of oncolytic viruses [11]. We analyzed induction of PKR and downstream eIF2 proteins by Western blot (Figure 3A). PKR phosphorylation was not increased in response to exposure to VSV-GFP in any of the cell lines. However, VSV-hIFNβ infection resulted in increased induction of PKR phosphorylation in all of the cell lines except H460, which expresses very low levels of PKR. Downstream phosphorylation of eIF2α was only seen in H460 and A549 upon exposure to VSV-hIFNβ. High levels of eIF2B-ε have been associated with permissiveness to VSV in cancer cells with an intact PKR/eif2α pathway [12], however there was no consistent pattern observed in these cell lines.

Several other pathways have been implicated in mediating tumor tropism of VSV including endoplasmic reticulum (ER) stress pathways [13], *k-ras* mutation [14], and cap-dependent protein translation [15]. IREα, a key ER stress protein, has been considered an inhibitor of *rhabdovirus* infection [13]; however, the only cell line to have increased expression of IREα was H2030, which was very sensitive to VSV-hIFNβ (Figure 3B). Likewise PERK, BIP, CHOP, Ero1-Lα and Calnexin expression, other important proteins in the ER stress pathway, were not markedly different in sensitive and resistant cell lines, suggesting that the ER stress pathway is not involved in mediating viral sensitivity. Taken together, it can be concluded that the type I IFN response is blunted in NSCLC cells and likely accounts for the tumor tropism of VSV. Virally produced IFNβ can still exert effects on the signaling pathway and induce interferon-stimulated genes such as STAT1 which is necessary for H838 cells to be resistant. JAK/STAT activation alone is not sufficient to impair viral replication as observed with A549, H2009, and H2030 cells.

### VSV has antitumor efficacy in human xenografts

**Figure 4:**
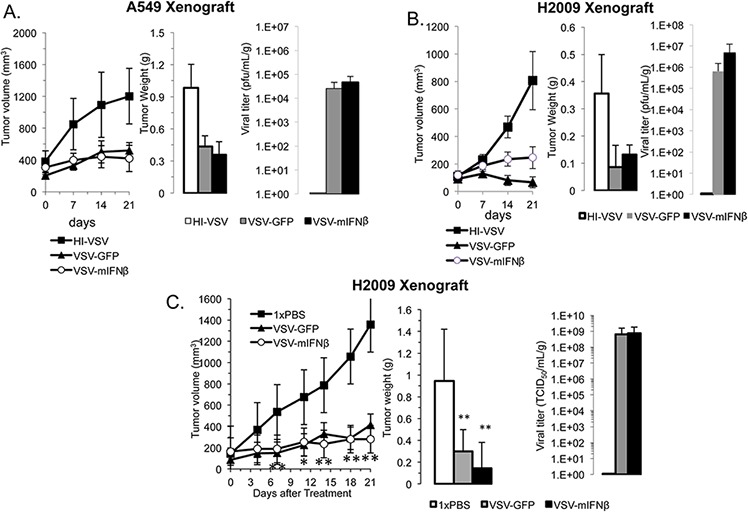
VSV has antitumor efficacy in human xenografts Nude mice bearing A549 **A.** and H2009 **B.** xenografts were treated with 6.6 × 10^8^ TCID50 heat-inactivated VSV (HI-VSV), VSV-GFP, or VSV-mIFNβ by intratumoral injection on days 0, 7, and 14. One week after the last injection (day 21), mice were sacrificed and tumors resected (*n* = 5 per group). Tumor volumes were measured with calipers in two dimensions (A and B, left). Tumors were weighed after sacrifice (A and B, middle). Viral titer was determined from resected tumors on day 21 (A and B, right). **C.** H2009 xenografts were treated with 1× PBS, 5 × 10^8^ TCID50 VSV-GFP or VSV-mIFNβ given on days 0, 2, and 4 followed by observation until day 21. Tumor volume was measured by calipers twice weekly (left). All mice were sacrificed on day 21, and resected tumors were weighed (middle) and assayed for viral titer on day 21 (right). Error bars indicate standard error of the mean (SEM), * indicates *p*-value < 0.05, ** indicates *p*-value < 0.001.

*In vivo* effects of VSV-GFP and VSV-mIFNβ on NSCLC tumors were tested in a nude mouse xenograft model using A549 and H2009 cell lines (Figure 4). Established tumors were treated by intratumoral injections of 6.6 × 10^8^ TCID50 on days 0, 7, and 14. On day 21, all mice were euthanized and tumors excised. Both VSV-GFP and VSV-mIFNβ showed antitumor activity in the A549 and H2009 xenograft models (Figure 4A & 4B). Tumor volume (Figure 4A and 4B, left) and tumor weights (Figure 4A and 4B, middle) after 21 days were lower in treated animals than control animals (*n* = 5), however these results did not achieve statistical significance. In both tumor models, live virus was recovered from tumors one week after the last viral injection, suggesting that further antitumor activity may have been observed with longer treatment (Figure 4A and 4B). In these experiments, there was no toxicity to the mice at the doses used. Their weights remained stable, and they did not show any signs of neurotoxicity during the experiment (data not shown). A similar experiment was done with H2009 xenografts in which tumors were injected on days 0, 2, and 4 with 5 × 10^8^ TCID50 VSV-GFP or VSV-mIFNβ followed by observation (Figure 4C). Tumor volume curves separated early and were significantly different between treated and untreated mice from day 7 onwards (*p* < 0.01 for each time point) (Figure 4C). Tumor weights were also significantly decreased upon sacrifice on day 21 (Figure 4C). Replicating virus was recovered from excised tumor tissue despite treatment 2 weeks earlier (Figure 4C, right). These *in vivo* data suggest that VSV is effective in immune deficient models of NSCLC.

### VSV-mIFNβ treatment results in improved survival in immune competent A/J mice bearing subcutaneous lung tumors

**Figure 5:**
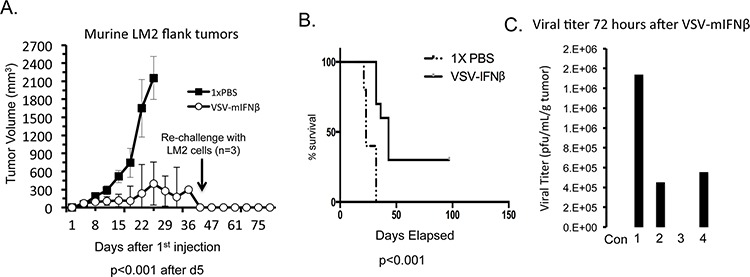
VSV-mIFNβ treatment of LM2 in immune competent A/J mice **A.** Mice (*n* = 10) were treated with intratumoral injections of 1× PBS or VSV-mIFNβ at a dose of 1.5 × 10^10^ TCID50 every other day for 3 doses. Estimate of tumor volume based on 2-dimensional measurements are shown. At day 45, 3 mice had no visible tumors and were re-challenged with 1 × 10^6^ LM2 cells injected in the flank. After 30 days, no tumors grew in these mice. **B.** Kaplan-Meier curve of survival of mice as defined as the date mice were sacrificed either because there tumors were larger than 1.5 cm^3^ or they had ulcerated tumor requiring sacrifice in accordance with ethical standards. Data were analyzed with log rank test and curves were significantly different (*p* < 0.001). **C.** A separate group of 5 mice were similarly treated with 1.5 × 10^10^ TCID50 VSV-mIFNβ and were sacrificed 72 hours later. Viral titers from resected tumors were determined using plaque assay. Four mice were treated with VSV-mIFNβ and one mouse was given PBS (Con). Error bars indicate standard deviation.

We next tested the effects of VSV-mIFNβ in an immune competent model. Syngeneic immune competent A/J mice were injected with 1 × 10^6^ murine LM2 cells, which are urethane-induced lung cancer cells [16]. All mice formed tumors within 11 days of injection. Because there was no toxicity in the nude mouse model, we increased the dose in the immune competent model to 1.5 × 10^10^ TCID50 given every other day for 3 doses. Tumor measurements were made once weekly, and when tumors either ulcerated through the skin or were larger than 1.5 cm^3^, mice were euthanized. Antitumor effects were seen very early with a clear separation in the tumor volume curve by day 5 (Figure 5A; *p* < 0.001). All control mice were sacrificed by day 19 either for reaching the tumor size endpoint or having ulcerated tumors. Thirty percent of treated mice had complete regression of tumors. The mice that had complete regression of tumors were rechallenged with 1 × 10^6^ LM2 cells in the opposite flank on day 33. None of the rechallenged mice developed tumors after 90 days of observation. This finding suggests the possibility that VSV-mIFNβ stimulates the development of immunologic memory to cancer cells. Survival of mice was estimated using the Kaplan-Meier method, and there was a statistically significant improvement in survival of the mice treated with VSV-mIFNβ (Figure 5B; *p* < 0.001). To measure viral titers, a separate cohort of mice were treated with the same dose of intratumoral VSV-mIFNβ and sacrificed 72 hours after the last treatment. Viral titers were recovered from 3 of 4 mice 72 hours after treatment (Figure 5C). Notably, none of the mice showed any signs of toxicity at any point during the study. In a syngeneic immune competent mouse model of NSCLC, intratumoral VSV-IFNβ treatment results in tumor regression and possible induction of antitumor immune response.

### Intratumoral injection of VSV-mIFNβ results in an abscopal immune response

**Figure 6:**
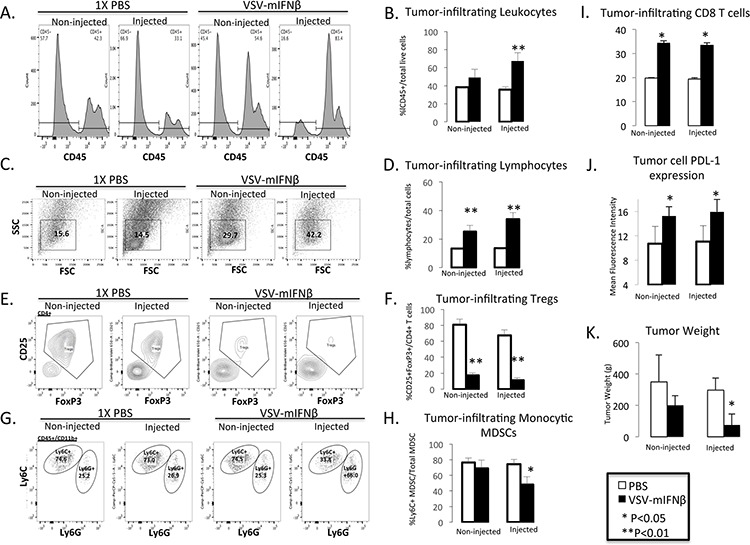
Flow cytometric analysis of tumor infiltrating leukocytes in injected and contralateral non-injected tumors after treatment with VSV-mIFNβ or 1× PBS **A.** Representative histograms of CD45+ leukocytes. **B.** Pooled data from *n* = 5 mice of total tumor infiltrating leukocytes. **C.** Representative scatter plot of lymphocyte population from VSV-mIFNβ-treated and control mice. FSC = Forward Scatter, SSC = Side scatter **D.** Pooled data from *n* = 5 mice of the total tumor infiltrating lymphocyte population. **E.** Representative Tumor infiltrating CD4+CD25+FoxP3+ T_reg_ cells. **F.** Pooled data from *n* = 5 mice of the percentage of tumor infiltrating T_reg_ cells. **G.** Representative histograms of tumor infiltrating monocytic MDSC (CD11b+Ly6ChiLy6G-) and polymorphonuclear MDSC (CD11b+Ly6CintLy6G+) cells. **H.** Pooled data of percentage of tumor infiltrating monocytic MDSC. **I.** Pooled data of percentage of tumor infiltrating CD8^+^ T cells. **J.** Pooled data of PDL-1 expression in CD45- tumor cells. Data are expressed as the Mean fluorescence intensity (PDL-1 expression/Isotype IgG). For each of the bar graphs, error bars indicate standard deviation. * denotes *p* < 0.05, ** denotes *p* < 0.001 comparing VSV-mIFNβ treated mice to PBS-treated mice. **K.** Tumor weights at the time of sacrifice 10 days after 1^st^ VSV-mIFNβ injection. Error bars indicate standard deviation.

Because the prior experiment demonstrated the possibility of an antitumor immune response, further experiments were done to try to characterize the immune response to intratumoral injection of VSV-mIFNβ. Using the same syngeneic lung cancer model, LM2, A/J mice were induced to form bilateral tumors. Once bilateral tumors formed, mice were given intratumoral injections of VSV-mIFNβ or 1× PBS into the right flank only on days 1, 3 and 5. The mice were sacrificed on day 10 and tumor-infiltrating leukocytes (TIL) were analyzed by flow cytometry (Figure 6). In mice treated with VSV-mIFNβ, both locally treated and opposite flank tumors showed an increase in (TILs) (Figure 6A–6D) coupled with a dramatic decrease in regulatory T cells (T_regs_) (Figure 6E and 6F). Also, CD8^+^ T cell infiltration was increased as well (Figure 6I). Furthermore, in the locally injected but not in distant tumors, there was a marked decrease in immune suppressive monocytic myeloid derived suppressor cells (MDSC) (Figure 6G & 6H). There was no significant change in NK cells or dendritic cell infiltration after intratumoral injection (data not shown). Though all mice were sacrificed at day 10, injected tumors were already significantly smaller in VSV-mIFNβ treated mice (Figure 6K). In mice treated with VSV-mIFNβ there was also a trend towards smaller tumors in the opposite flank as well though this did not reach statistical significance. VSV-mIFNβ also resulted in a significant increase in PDL-1 expression on tumor cells in both injected and non-injected tumors after VSV-mIFNβ treatment (Figure 6J). Taken together, these data strongly suggest that intratumoral injection of VSV-mIFNβ results in a systemic antitumor immune response and immunologic memory. The observation of increased tumor cell PDL-1 expression may limit the full potential of antitumor immunity and might lead ultimately to tumor progression, though this is speculative.

## DISCUSSION

The present study indicates a potential role of VSV-IFNβ as a therapeutic agent for NSCLC. VSV-IFNβ has begun clinical testing in hepatoma, and the successful completion of this first in human study will be crucial for further development of VSV-IFNβ for other tumor types. Our *in vitro* and *in vivo* data show proof-of-principle that VSV-IFNβ has oncolytic effects on human and murine lung tumors. Our data in immune competent mice are particularly intriguing, as they suggest not only a direct oncolytic effect of the tumor, but also the potential for developing anti-tumor immunity. There have been published reports of preclinical data in immune competent models of myeloma, melanoma, and mesothelioma in which VSV-IFNβ infection results in CD8^+^ mediated tumor cytotoxicity [5, 17, 18]. While the data presented do not delineate a mechanism of anti-tumor immunity, the marked decrease in T_regs_ and increased CD8^+^ T cell infiltration are likely to be important in the ultimate development of immunologic memory. How much of the observed effect is dependent upon the viral transgene, IFNβ, is not clear. Previous data indicated that VSV alone (not-expressing IFNβ) induced infiltration of MDSC [19]. In contrast, we report here that MDSCs, particularly immune suppressive monocytic MDSC are reduced after VSV-IFNβ treatment [20, 21]. In mesothelioma, the antitumor effect of VSV-IFNβ was partly dependent on biologically active IFNβ [5]. There are data to suggest that the type I IFN response may be a necessary signal for differentiation of CD8^+^ T cells to memory T cells [22–24]. Taken together, we believe that the IFNβ transgene is quite likely important for the immune-stimulation following VSV-IFNβ treatment. Ongoing studies are investigating further the mechanism by which VSV-IFNβ stimulates a therapeutic immune response in NSCLC animal models.

The increased PDL-1 expression induced by VSV-IFNβ therapy is also intriguing, particularly in light of the recent clinical development of immune checkpoint blockade for NSCLC. PD-1 inhibitors, nivolumab and pembrolizumab, have now demonstrated impressive antitumor properties in NSCLC patients leading to recent FDA approval of nivolumab [25–28]. While response rates are in the range of 10–25% to these drugs, the response is higher in patients with high tumor expression of PDL-1. Moreover, the presence of tumor-infiltrating CD8^+^ T cells appear to be required for response to PD-1 blockade [29]. Therefore, our data suggest that VSV-IFNβ could be synergistic with PD-1 or PDL-1 inhibitors. Recently, intratumoral Newcastle disease virus (NDV) has been shown to sensitize murine melanoma to immune checkpoint blockade, highlighting the promise of this approach [30].

The lack of response in IFN signaling after infection with VSV-GFP indicate that the type I IFN response to viral infection is indeed defective in many NSCLC cell lines as has been previously demonstrated [9, 31]. The differential effect of VSV-GFP and VSV-hIFNβ on H838 cells strongly suggested that a component of the IFNβ pathway is responsible for resistance. This notion is further supported by inhibition of JAK/STAT signaling, which completely sensitizes H838 cells to VSV-hIFNβ oncolysis. However, it is difficult to suggest a predictive biomarker as IFN signaling appeared to be similar amongst most of the cell lines tested. H460 which was the most sensitive to VSV-hIFNβ was markedly abnormal in a variety of IFN signaling proteins, however, H2009, A549, and H2030 displayed similar IFN signaling characteristics to H838 despite retaining sensitivity to VSV-hIFNβ. Therefore, different components of the IFN pathway might be defective in sensitive cell lines precluding the use of a single biomarker. A type I IFN signature has been previously applied to patients receiving IFNβ therapy for multiple sclerosis, and perhaps such a tool could be identified that would predict resistance to VSV-hIFNβ [32]. Such studies could be critical as VSV-hIFNβ undergoes further clinical development.

Despite evidence from other models that inhibition of protein translation would inhibit oncolytic virotherapy, our data suggest otherwise. Phosphorylation of eIF2α (presumed to inhibit translation) was increased upon VSV-hIFNβ only in H460 and A549, which were the most permissive for viral replication indicating that this phosphorylation was insufficient to impair viral replication. Perhaps the phosphorylation of eIF2α occurs too late to stop viral translation or the signal is not strong enough. On the contrary, the resistant cell line, H838, was not induced to phosphorylate eIF2α following VSV-hIFNβ infection despite being resistant to VSV-hIFNβ. Therefore, eIF2α phosphorylation is not required for resistance to VSV-hIFNβ infection.

In the above experiments, VSV-mIFNβ was delivered by intratumoral injection. For NSCLC, VSV-hIFNβ would be ideally delivered intravenously to patients as the majority of patients have metastatic disease. Recent work in mouse models has demonstrated that VSV is sequestered in lymph tissue upon IV delivery resulting in much less of the therapeutic reaching tumor sites even in mice without circulating anti-VSV neutralizing antibodies [33]. As a result, alternate approaches to delivering VSV systemically may need to be employed. Cyclophosphamide can be given to mice to reduce neutralizing antibody titers and has been shown to increase the half-life of circulating VSV [34], however in some models cyclophosphamide can have a negative impact on antitumor activity of VSV [19]. Utilizing viral carriers also has promise for virotherapy and has been applied to VSV therapy successfully in animal models [35]. The data presented in this manuscript provide a rationale for using intratumoral injection of VSV-IFNβ to drive a systemic immune response and thereby avoid the pitfalls of systemic treatment. The current phase I trial of VSV-hIFNβ utilizes intratumoral injections into liver tumors with imaging guidance. Thus, data from this phase I trial could be translated to CT-guided intratumoral injections of lung tumors in order to have a local effect, but also potentially a systemic immune effect. The recent FDA approval of talimogene laherparepvec by intratumoral injection highlights the potential of using intratumoral injections for systemic disease [36].

In summary, the work presented here demonstrates the potential of VSV-IFNβ as a novel therapeutic agent for treatment of NSCLC. Further studies of VSV-IFNβ, particularly with regards to the immunostimulatory properties, are currently being explored to optimize clinical translation for patients with NSCLC.

## MATERIALS AND METHODS

### Cell lines and cell culture

The medium for NSCLC cell lines, H460, A549, H2009 and H2030 was RPMI 1640 (Gibco, Life Technologies) containing 10% calf serum (Biofluids). H838 cells were grown in RPMI 1640 supplemented with 10% calf serum, 10 mM HEPES, 1 mM sodium pyruvate, 4.5 g/L glucose, and 1.5 g/L sodium bicarbonate. Beas2B cells, non-transformed, immortalized human bronchial epithelial cells, were maintained in keratinocyte-serum free medium supplemented with recombinant epidermal growth factor and bovine pituitary extract (Life Technologies). Cell lines were authenticated by short tandem repeat profiling performed by an independent laboratory (Johns Hopkins Cell Authentication Facility). African green monkey kidney Vero cells (CCL-81) were maintained in DMEM supplemented with 5% calf serum. Lewis lung carcinoma cells were grown in Dulbecco's modified Eagle's medium supplemented with 10% calf serum. The medium for the murine (A/J mouse) urethane induced lung cancer (LM2) [16] cells was minimal essential medium-alpha supplemented with L-glutamine and 10% calf serum. All cell lines were from the American Tissue Culture Collection except for LM2, which was kindly provided by the laboratory of Dr. Alvin M. Malkinson, Department of Pharmaceutical Sciences, University of Colorado, Denver, Colorado. Cells were maintained at 37°C in 5% CO2.

### Viruses

VSV (Indiana Strains) were engineered to produce green fluorescence protein (VSV-GFP) or human or mouse interferon-β (hIFNβ or mIFNβ, respectively) at the Mayo Clinic Viral Vector Core Facility (Rochester, MN) as previously described [6, 18]. All viral stocks were grown in Vero cells and titered using limiting dilution assays or standard plaque assay on Vero cells.

### Cell lysate preparation following VSV treatment

Human NSCLC (H460, A549, H838, H2009 and H2030) and immortalized Beas2B cells (6 × 10^6^) were seeded onto 15 cm plates and grown in their appropriate medium. Following overnight incubation cells were rinsed with Opti-MEM (Gibco, Life Technologies) and then incubated with either only Opti-MEM or Opti-MEM containing an MOI equaling 0.1 of VSV-gfp or VSV-hIFNβ. The cells were harvested 24 hours later from the plates by scraping after washing once with PBS (phosphate buffered saline), after which cells were collected by centrifugation (14K rpm, 14 seconds), washed again with ice cold PBS followed by another round of centrifugation and resuspended in 1× Cell Lysis Buffer (Cell Signaling) containing 1 mM phenylmethanesulfonyl fluoride (Sigma-Aldrich). The protein concentrations were determined by Bradford assay and stored at −80°C.

### Western blot analysis

Protein samples were separated by 10% SDS-PAGE (polyacrylamide gel electrophoresis) or by 8–15% gradient gels. Following protein transfer to PVDF the membranes were blocked in 5% non-fat dry milk for 1 hour at room temperature in Tris-buffered saline-Tween (TBST: 0.15 M NaCl; 0.01 M Tris-HCl, pH 7.6; 0.05% Tween 20). The membranes were then incubated for 1 hour at ambient temperature or overnight at 4°C with the chosen primary antibody. The primary antibodies employed from Cell Signaling were rabbit α-STAT1 [#9172], rabbit α-Phospho-STAT1 (Tyr701) antibody [#9171], mouse α-STAT3 [#9139], rabbit α-Phospho-STAT3 (Tyr705) antibody [#9145], rabbit α-PKR antibody, rabbit α-PERK antibody [#3192], rabbit α-Ero1-Lα antibody [#3264], mouse α-CHOP antibody [#2895], rabbit α-Calnexin antibody [#2679], rabbit α-IRE1α antibody [#3192] and rabbit α-BiP antibody [#3177] each at a 1:1000 dilution. Other primary antibodies utilized were rabbit α-p48 antibody [sc-496] from Santa Cruz Biotechnology at 1:500 dilution and mouse α-β-actin [A1978] (Sigma) at a 1:10,000 dilution. Preceding and following incubation with the appropriate horseradish peroxidase labeled secondary antibody, the blots were washed three times for 5 minutes in TBST. Detection was performed utilizing ECL Plus Western Blotting System (Amersham Biosciences) to visualize the bands of interest.

### *In vitro* and *in vivo* viral titer

For *in vitro* titers, cells were treated as above with an MOI of 0.1 and medium samples removed at 24, 48 and 72 hours and stored at −80°C. For *in vivo* titer determinations, flank tumors were aseptically removed, frozen and stored at −80°C. Portions of each tumor analyzed were weighed and placed in a gentleMACS M tube containing 2 mL of PBS on ice. Samples were homogenized using a gentleMACS dissociator (Miltenyl Biotec Inc.) following the manufacture's instructions. Samples were clarified by centrifugation (3000 × g, 5 min.). Titers were measured by infection of Vero cells (7000 cells/well) in 96-well plates with 1:5 serial dilutions of medium samples containing VSV. The tissue culture infective dose 50 (TCID50) were assessed employing the Spearman and Karber method [37]. The TCID50 for the tumor homogenates was normalized to volume (mL) and tumor weight (g) and expressed as the mean +/− SD. For some of the tumor samples the titer was measured utilizing a viral plaque assay to determine plaque-forming units per mL per gram tumor (pfu/mL/g). Vero cells (6 × 10^5^/well) were seeded onto 6 well plates and inoculated with serial dilutions in triplicate of tumor homogenates, then overlayed with (0.5%) agarose-DMEM medium mixture. Twenty four hours later cells were fixed with a 3:1 ratio of a methanol-acetic acid mixture, agarose overlay removed and cells stained with coomassie brilliant blue (Sigma). Plaque numbers were counted, normalized to volume (mL) and tumor weight (g) and expressed as the mean +/− SD [38].

### Cell viability assays

Cells were seeded as triplicate sets into 6-well plates with 150,000 cells per well for Beas2B, H460, A549, H838, H2009 H2030, LLC and 200,000 cells per well for LM2. Following overnight incubation cells were rinsed with Opti-MEM (Gibco, Life Technologies) and then incubated with Opti-MEM containing the indicated multiplicity of infection (MOI) of VSV-gfp or VSV-IFNβ for two hours. LLC and LM2 were treated with VSV that secreted the murine form of IFNβ (VSV-mIFNβ), while the human NSCLC cells were treated with VSV that secreted the human form (VSV-hIFNβ). Fresh medium was then added and 72 hours later, the cell number determined by counting viable cells after exposure to trypan blue. Cell survival is shown as a percentage of untreated cells.

### Combination VSV-hIFNβ and ruxolitinib

Five thousand H460 or H838 cells were seeded onto 96-well plates in triplicate. After overnight incubation, cells were treated with the indicated concentrations of Ruxolitinib (Selleck Chemicals), VSV-mIFNβ at the specified multiplicity of infection (MOI), or with both agents. Control cells were treated with identical concentration of vehicle (0.4% DMSO). Cell viability was determined following seventy-two hour incubation employing Cell Counting Kit-8 (Dojindo Molecular Technologies) following manufacturers protocol. Cell survival was normalized to vehicle-treated cells. Experiments were performed in triplicate. In a parallel experiment performed as just described, cell supernatant samples were harvested from each well 24 and 48 h following infection of both H460 and H838 cell lines with and without ruxolitinib treatment and stored at −80°C for determination of viral titers.

### IFN-β ELISA

NSCLC Cells were treated as described above for the cell viability assay and culture medium harvested and stored at −80°C for cells subjected to VSV at an MOI of 0.1 for 48 hours. The level of Human IFN-β was quantified in the medium by employing VeriKine human IFNβ ELISA kit according to manufacturer's instructions (PBL Assay Science). Positive and negative controls are included in the kit and all samples were done in triplicate.

### Flow cytometry

A/J mice were sacrificed 5 days after last treatment. Tumors and spleens were homogenized using a mouse Tumor Dissociation Kit (Miltenyi Biotec) according to manufacturer's instructions. Cell suspensions were stained according to manufacturer instructions. Intracellular staining for FoxP3 was performed using the Foxp3/Transcription Factor Staining Buffer Set (eBioscience: 00-5523).

The stained cells were analyzed on an LSRII flow cytometer (BD Biosciences). Cells were gated and identified as follows: CD8 T-cells (CD8^+^), NK cells (CD8^−^, CD4^−^, CD49b^+^), T-regulatory cells (CD4^+^, CD25^+^, FoxP3^+^), monocytic MDSCs (CD45^+^, CD11b^+^, Ly6C^Hi^), granulocytic MDSCs (CD45^+^, CD11b^+^, Ly6G^Hi^), and dendritic cell (CD11b^+^, CD11c^+^, CD8^+^). Antibody conjugates were purchased from BioLegend: αCD8/FITC (100705), αCD4/PerCP-Cy5.5 (100539), αCD49b/PE/Cy7 (108921), αCD45.2/PE (109808), αCD11b/BV650 (101239), αPDL1/PE-Cy7 (124313), αPDL1/PE-Cy7 isotype (400617), αCD25/BV650 (102038), BD Pharmingen: αLy6C/PerCP/Cy5.5 (560525) and αLy6G/AF700 (561236), and eBioscience: FoxP3/AF700 (56-5773-80). Dead cells were excluded from analysis using Fixable Viability Dye eFluor 780 (eBioscience 65-0865-14).

### Animal experiments

2.5 × 10^6^ A549 or 2.5 × 10^6^ H2009 (Figure 4B) or 2 × 10^6^ (Figure 4C) H2009 cells in 0.1 mL 1× PBS were injected into the flanks of 4–6 week old nude mice (nu/nu; NCI). When tumors were 0.5 cm^3^ the mice received intratumoral injections once weekly for three weeks or every other day for three treatments of heat inactivated VSV-mIFN-β (*n* = 5), VSV-gfp (*n* = 5) or VSV-mIFN-β (*n* = 5) at 5 × 10^8^ or 6.6 × 10^8^ TCID50 in a 0.1 mL volume. Tumor sizes were measured on the indicated days after the start of treatment. The long (D) and the short diameter (d) were measured with a digital caliper (Fisher). Tumor volume (mm^3^) was determined as V = d^2^ × D × 0.5. Mice were sacrificed on day 21 following the start of treatment and the excised tumors were weighed. For the syngeneic model, 1 × 10^6^ LM2 cells were injected into either unilateral or bilateral flanks of 6 week old A/J (The Jackson Laboratory, Maine, USA) mice using a 25 gauge needle. These mice were treated with intratumoral injections of 1× PBS (*n* = 10) or VSV-mIFN-β (*n* = 10) at 1.5 × 10^10^ TCID50 in a 0.1 mL volume every other day for three total treatments. Mice were sacrificed when tumors reached 1.5 cm^3^ or if tumors ulcerated. Mice given bilateral flank tumors were injected unilaterally as above, however, these mice were sacrificed 5 days after last intratumoral injection and tumors were excised for further analysis. All procedures involving animals were performed according to guidelines of the Institutional Animal Care and Use Committee of the University of Minnesota (Protocol # 1309-30941A).

### Statistical analysis

*In vitro* experiments were done in triplicate. Data are expressed as a mean and error bars indicate either standard deviation or standard error of the mean as indicated in the figure legends. Statistical analysis of *in vitro* and *in vivo* data were done using 2-sided paired *t*-tests with *p* value < 0.05 taken as significant. Survival of mice was estimated using the Kaplan-Meier method. Kaplan-Meier curves were generated in GraphPad Prism software (v. 6.0). Statistical analysis of the differences between control and treated groups were performed using the log-rank test. A *p* value < 0.05 was taken as significant.

## References

[1] Patel MR, Kratzke RA (2013). Oncolytic virus therapy for cancer: the first wave of translational clinical trials. Transl Res.

[2] Russell SJ, Peng KW, Bell JC (2012). Oncolytic virotherapy. Nat Biotechnol.

[3] Li Q, Wei YQ, Wen YJ, Zhao X, Tian L, Yang L, Mao YQ, Kan B, Wu Y, Ding ZY, Deng HX, Li J, Luo Y (2004). Induction of apoptosis and tumor regression by vesicular stomatitis virus in the presence of gemcitabine in lung cancer. Int J Cancer.

[4] Barber GN (2004). Vesicular stomatitis virus as an oncolytic vector. Viral Immunol.

[5] Willmon CL, Saloura V, Fridlender ZG, Wongthida P, Diaz RM, Thompson J, Kottke T, Federspiel M, Barber G, Albelda SM, Vile RG (2009). Expression of IFN-beta enhances both efficacy and safety of oncolytic vesicular stomatitis virus for therapy of mesothelioma. Cancer research.

[6] Obuchi M, Fernandez M, Barber GN (2003). Development of recombinant vesicular stomatitis viruses that exploit defects in host defense to augment specific oncolytic activity. Journal of virology.

[7] Jenks N, Myers R, Greiner SM, Thompson J, Mader EK, Greenslade A, Griesmann GE, Federspiel MJ, Rakela J, Borad MJ, Vile RG, Barber GN, Meier TR (2010). Safety studies on intrahepatic or intratumoral injection of oncolytic vesicular stomatitis virus expressing interferon-beta in rodents and nonhuman primates. Hum Gene Ther.

[8] Faul EJ, Lyles DS, Schnell MJ (2009). Interferon response and viral evasion by members of the family rhabdoviridae. Viruses.

[9] Stojdl DF, Lichty BD, tenOever BR, Paterson JM, Power AT, Knowles S, Marius R, Reynard J, Poliquin L, Atkins H, Brown EG, Durbin RK, Durbin JE (2003). VSV strains with defects in their ability to shutdown innate immunity are potent systemic anti-cancer agents. Cancer Cell.

[10] Lee J, Jung HH, Im YH, Kim JH, Park JO, Kim K, Kim WS, Ahn JS, Jung CW, Park YS, Kang WK, Park K (2006). Interferon-alpha resistance can be reversed by inhibition of IFN-alpha-induced COX-2 expression potentially via STAT1 activation in A549 cells. Oncol Rep.

[11] Balachandran S, Barber GN (2007). PKR in innate immunity, cancer, and viral oncolysis. Methods Mol Biol.

[12] Balachandran S, Barber GN (2004). Defective translational control facilitates vesicular stomatitis virus oncolysis. Cancer Cell.

[13] Mahoney DJ, Lefebvre C, Allan K, Brun J, Sanaei CA, Baird S, Pearce N, Gronberg S, Wilson B, Prakesh M, Aman A, Isaac M, Mamai A (2011). Virus-tumor interactome screen reveals ER stress response can reprogram resistant cancers for oncolytic virus-triggered caspase-2 cell death. Cancer Cell.

[14] Balachandran S, Porosnicu M, Barber GN (2001). Oncolytic activity of vesicular stomatitis virus is effective against tumors exhibiting aberrant p53, Ras, or myc function and involves the induction of apoptosis. J Virol.

[15] Connor JH, Lyles DS (2002). Vesicular stomatitis virus infection alters the eIF4F translation initiation complex and causes dephosphorylation of the eIF4E binding protein 4E-BP1. J Virol.

[16] Malkinson AM, Dwyer-Nield LD, Rice PL, Dinsdale D (1997). Mouse lung epithelial cell lines—tools for the study of differentiation and the neoplastic phenotype. Toxicology.

[17] Naik S, Nace R, Barber GN, Russell SJ (2012). Potent systemic therapy of multiple myeloma utilizing oncolytic vesicular stomatitis virus coding for interferon-beta. Cancer Gene Ther.

[18] Diaz RM, Galivo F, Kottke T, Wongthida P, Qiao J, Thompson J, Valdes M, Barber G, Vile RG (2007). Oncolytic immunovirotherapy for melanoma using vesicular stomatitis virus. Cancer research.

[19] Willmon C, Diaz RM, Wongthida P, Galivo F, Kottke T, Thompson J, Albelda S, Harrington K, Melcher A, Vile R (2011). Vesicular stomatitis virus-induced immune suppressor cells generate antagonism between intratumoral oncolytic virus and cyclophosphamide. Mol Ther.

[20] Shirota Y, Shirota H, Klinman DM (2012). Intratumoral injection of CpG oligonucleotides induces the differentiation and reduces the immunosuppressive activity of myeloid-derived suppressor cells. J Immunol.

[21] Zhu B, Bando Y, Xiao S, Yang K, Anderson AC, Kuchroo VK, Khoury SJ (2007). CD11b+Ly-6C(hi) suppressive monocytes in experimental autoimmune encephalomyelitis. J Immunol.

[22] Curtsinger JM, Schmidt CS, Mondino A, Lins DC, Kedl RM, Jenkins MK, Mescher MF (1999). Inflammatory cytokines provide a third signal for activation of naive CD4+ and CD8+ T cells. J Immunol.

[23] Curtsinger JM, Valenzuela JO, Agarwal P, Lins D, Mescher MF (2005). Type I IFNs provide a third signal to CD8 T cells to stimulate clonal expansion and differentiation. J Immunol.

[24] Valenzuela J, Schmidt C, Mescher M (2002). The roles of IL-12 in providing a third signal for clonal expansion of naive CD8 T cells. J Immunol.

[25] Rizvi N, Garon EB, Patnaik A, Gandhi L (2014). Safety and clinical activity of MK-3475 as initial therapy in patients with advanced non-small cell lung cancer (NSCLC).

[26] Rizvi N, Chow L.Q, Dirix L.Y, Gettinger S.N, Gordon M.S (2014). Clinical trials of MPDL3280A (anti-PDL1) in patients (pts) with non-small cell lung cancer (NSCLC).

[27] Rizvi NA, Mazieres J, Planchard D, Stinchcombe TE, Dy GK, Antonia SJ, Horn L, Lena H, Minenza E, Mennecier B, Otterson GA, Campos LT, Gandara DR (2015). Activity and safety of nivolumab, an anti-PD-1 immune checkpoint inhibitor, for patients with advanced, refractory squamous non-small-cell lung cancer (CheckMate 063): a phase 2, single-arm trial. The Lancet Oncology.

[28] Garon EB, Rizvi NA, Hui R, Leighl N, Balmanoukian AS, Eder JP, Patnaik A, Aggarwal C, Gubens M, Horn L, Carcereny E, Ahn MJ, Felip E (2015). Pembrolizumab for the Treatment of Non-Small-Cell Lung Cancer. N Engl J Med.

[29] Taube JM, Klein A, Brahmer JR, Xu H, Pan X, Kim JH, Chen L, Pardoll DM, Topalian SL, Anders RA (2014). Association of PD-1, PD-1 ligands, and other features of the tumor immune microenvironment with response to anti-PD-1 therapy. Clin Cancer Res.

[30] Zamarin D, Holmgaard RB, Subudhi SK, Park JS, Mansour M, Palese P, Merghoub T, Wolchok JD, Allison JP (2014). Localized oncolytic virotherapy overcomes systemic tumor resistance to immune checkpoint blockade immunotherapy. Science translational medicine.

[31] Li Q, Tainsky MA (2011). Epigenetic silencing of IRF7 and/or IRF5 in lung cancer cells leads to increased sensitivity to oncolytic viruses. PLoS One.

[32] van Baarsen LG, Vosslamber S, Tijssen M, Baggen JM, van der Voort LF, Killestein J, van der Pouw Kraan TC, Polman CH, Verweij CL (2008). Pharmacogenomics of interferon-beta therapy in multiple sclerosis: baseline IFN signature determines pharmacological differences between patients. PLoS One.

[33] Tesfay MZ, Ammayappan A, Federspiel MJ, Barber GN, Stojdl D, Peng KW, Russell SJ (2014). Vesiculovirus neutralization by natural IgM and complement. J Virol.

[34] Peng KW, Myers R, Greenslade A, Mader E, Greiner S, Federspiel MJ, Dispenzieri A, Russell SJ (2013). Using clinically approved cyclophosphamide regimens to control the humoral immune response to oncolytic viruses. Gene Ther.

[35] Power AT, Wang J, Falls TJ, Paterson JM, Parato KA, Lichty BD, Stojdl DF, Forsyth PA, Atkins H, Bell JC (2007). Carrier cell-based delivery of an oncolytic virus circumvents antiviral immunity. Mol Ther.

[36] Andtbacka RH, Kaufman HL, Collichio F, Amatruda T, Senzer N, Chesney J, Delman KA, Spitler LE, Puzanov I, Agarwala SS, Milhem M, Cranmer L, Curti B, Lewis K, Ross M, Guthrie T (2015). Talimogene Laherparepvec Improves Durable Response Rate in Patients With Advanced Melanoma. J Clin Oncol.

[37] Hierholzer JCaK R.A (1996). Virus isolation and quantification.

[38] Diallo JS, Vaha-Koskela M, Le Boeuf F, Bell J (2012). Propagation, purification, and *in vivo* testing of oncolytic vesicular stomatitis virus strains. Methods Mol Biol.

